# Artificial Intelligence Inheriting the Historical Crisis in Psychology: An Epistemological and Methodological Investigation of Challenges and Alternatives

**DOI:** 10.3389/fpsyg.2022.781730

**Published:** 2022-03-10

**Authors:** Mohamad El Maouch, Zheng Jin

**Affiliations:** ^1^Henan International Joint Laboratory of Psychological Data Science, Zhengzhou Normal University, Zhengzhou, China; ^2^Department of Psychology, University of California, Davis, Davis, CA, United States

**Keywords:** psychology, methodology, Vygotsky, crisis, cultural-historical activity theory, contradiction-based meanings, epistemology, artificial intelligence

## Abstract

By following the arguments developed by Vygotsky and employing the cultural-historical activity theory (CHAT) in addition to dialectical logic, this paper attempts to investigate the interaction between psychology and artificial intelligence (AI) to confront the epistemological and methodological challenges encountered in AI research. The paper proposes that AI is facing an epistemological and methodological crisis inherited from psychology based on dualist ontology. The roots of this crisis lie in the duality between rationalism and objectivism or in the mind-body rupture that has governed the production of scientific thought and the proliferation of approaches. In addition, by highlighting the sociohistorical conditions of AI, this paper investigates the historical characteristics of the shift of the crisis from psychology to AI. Additionally, we examine the epistemological and methodological roots of the main challenges encountered in AI research by noting that empiricism is the dominant tendency in the field. Empiricism gives rise to methodological and practical challenges, including challenges related to the emergence of meaning, abstraction, generalization, the emergence of symbols, concept formation, functional reflection of reality, and the emergence of higher psychological functions. Furthermore, through discussing attempts to formalize dialectical logic, the paper, based on contradiction formation, proposes a qualitative epistemological, methodological, and formal alternative by using a preliminary algorithmic model that grasps the formation of meaning as an essential ability for the qualitative reflection of reality and the emergence of other mental functions.

## Introduction

Artificial intelligence has developed dramatically during the 21st century in almost all civil and military domains, resulting in a “threat” of human replacement. However, for many, such a feeling overestimates artificial intelligence (AI)’s capabilities because AI is still at the stage of artificial narrow intelligence (ANI) and not at the stage of human-like (or even animal-like) artificial general intelligence (AGI). This gap reveals the dichotomy between *weak* and *strong* AI (see e.g., Searle’s, 1980; Ekbia, 2008; Lu et al., 2021). In reality, numerous crucial challenges confront the development of AI, such as the challenges regarding the abilities of abstraction and generalization, the emergence of meanings/semantics and symbols, the functional reflection of reality, active learning and adaptation, and hardware-related problems.

The list above is a sample of inflation in the philosophical and psychological debate. The inflation is derived from “unsolved” epistemological and ontological questions such as self-consciousness, the nature of the mind, mind-body duality, the problems of meaning and knowledge production, etc. The inflation is derived as well from new trends, e.g., trends in artificial psychology, AI-related ethics, law, existential studies, and effects on the contemporary psyche (see e.g., Collins and Smith, 1988; Cummins and Pollock, 1991; Dennett, 1997; Turkle, 2005; Carter, 2007; Geraci, 2007; Wang, 2007; Hildt, 2019; Abraham, 2021; Thompson, 2021). Inflation refers to the fact that AI investigates and empirically tests both philosophy and psychology. AI combines tendencies toward abstraction (in philosophy) and explicit particularity (in psychology) (Dennett, 2017), hence emphasizing more starkly the intrinsic tensions of modernity, e.g., the tension between mind and body (Ekbia, 2008).

Therefore, despite the significant success, the gap between AI and natural (animal- or human-like) intelligence calls for collaboration among philosophy, psychology (including neuropsychology), and AI research (e.g., Sloman, 2014). AI needs philosophy (e.g., Masís, 2014) because AI does not have to *reinvent the wheels every few days* (see Dennett, 2017, p. 137). However, numerous obstacles constrain this desired collaboration. Some obstacles are rooted in current socio-historical conditions in science, academia, and the production of thoughts. Examples of these conditions are the institutional organization and educational systems, funding policies, researchers’ motivations, commercialization requirements, and the economization trends of neoliberalism, in addition to the trend toward dephilosophication in academia and science (see e.g., Ekbia, 2008; Al Chawk, 2011; Berman, 2014; Hoffman, 2017). In our opinion, the crucial obstacles are rooted in the ontological, epistemological, and methodological state governing each domain, i.e., philosophy, psychology, and AI. Briefly, philosophy and psychology historically had their own internal “unsolved” debate even before the development of AI. Therefore, collaboration only shifts the debate into the context of AI. This fact is why the outcomes of such invitations remain an open-ended discussion with general suggestions, but to which no methodological tools or experimental models have been introduced.

Despite the unsolved hard problems, the tension in AI has provided *tested* proofs of the necessary principles of intelligence and mind: a mind must be adaptive and have open-development characteristics, it must be rooted in needs and desires, being situated in and dependent on the environment through the sensorimotor system. Also, the mind must have the ability to abstract and generalize, and it must be able to grasp the semantics and meanings of phenomena. Furthermore, the mind must be able to represent causality, it must be active in terms of learning and engaging with components of the environment, and it must have narrative and agency ability. In addition, the mind must be able to adjust its internal representations of the world (usually called the frame problem), it must have the ability to interpret (inner-self), and to ground its representations in real-world experience in a dynamically structured way, and the components of this whole system have to be synthesized and fused, among other requirements (see e.g., Carter, 2007; Ekbia, 2008; Dennett, 2017).

Underlying this debate is the traditional ontological question in philosophy concerning *the origin of the mind* and the epistemological question of *how the mind knows reality (and can it do so at all)?* AI shares with “the traditional epistemology the status of being a most general, most abstract asking of the top-down question: how is knowledge possible?” (Dennett, 2017, p. 122). The underlying factors in this debate are the question of mind-body or mental-physical (thought-matter) duality and the connection between those elements and that of how the *subjective* (and higher mental states) appears from the *objective* (experience). Furthermore, AI is considered to be the modern inheritor of longstanding quests in philosophy and the history of humanity (Van der Veer and Valsiner, 1991; Ekbia, 2008).

In summary, AI has reproduced and clarified philosophical and psychological problems based on the repetitive historic regularity that governs the development and replacement of scientific ideas according to the objective demands of the phenomena under investigation (see Vygotsky, 1997). These demands are, in our case, the shared subject of matter of psychology and AI. Therefore, progress in AI not only requires cooperation among philosophy, psychology, and AI, as mainstream invitations have suggested but also requires that we consider the philosophical and psychological debates to be the sources of the impasse. This consideration exists because philosophy and psychology themselves stand at a historical impasse. As representatives of this impasse, we find statements regarding issues such as *the mystery of consciousness* that is *yet to be conceived* and *far from being understood* or claims that consciousness is a *black hole* or that there are *still no answers to the posed questions.* Other researchers have considered consciousness to be a negative reflection of brain processes or brain hallucinations and an illusion that cannot be grasped through the sciences but only through certain religious and contemplative practices (see e.g., O’Rourke, 1993; Taylor, 2000; Chella and Manzotti, 2011; Vacariu, 2011; Carruthers, 2017; Oakley and Halligan, 2017; Seth, 2017; Varela et al., 2017; Lu et al., 2021). Some researchers have suggested that the mind-body problem is a pseudoproblem (e.g., Vacariu, 2011)! These views are not ontologically and epistemologically new. Instead, they are modern reproductions of previous historical positions. However, these views condense the latent state of impasse in both the field of philosophy and that of psychology. This is why, since the early nineties, there has “been relatively little movement in the philosophical debate despite the terrific advances within cognitive science and other AI-related fields” (Estrada, 2014, p. 59). Therefore, due to a lack of answers and against the wishes of Dennett (2017), AI is obliged to reinvent itself as an intense and proliferated research area, a point which references the already-invented wheel in philosophy and psychology, as noted by Ekbia (2008). However, we assume that the shortcomings in this context derive from the mainstream approaches to philosophy and psychology, and one can still find aid in marginalized or not fully investigated approaches.

Therefore, alongside Ekbia’s (2008) extensive *con textual* social-economical-theoretical-technical investigations concerning the development of AI, we maintain that it is crucial to reflect critically on the ongoing debate and to evaluate the challenges by reading between the lines to identify the historical position of the debate, including latent reference to its not-fully discovered legacy. The attempt by Ekbia is informed by the previous critical legacy in and around the field, including figures such as McDermott (1976, 1987), Searle (1984), Hofstadter (1985, 1995), Woolgar (1985, 1989), Winograd and Flores (1986), Suchman (1987, 2006), Collins (1990), Dennett (1991), Smith (1991, 1996), Dreyfus (1992, 2014), Agre (1997, 2002), Edwards (1997), Forsythe (2001). In summary, we maintain that the debate, including competing schools and approaches, is the effect of what Vygotsky called *the historical crisis in psychology* (Vygotsky, 1997). By revising Vygotsky’s century-old text, one can discover nearly identical main tendencies to those that govern the present debate and early contemplations concerning how the debate may develop. That text served as a prelude to the later work of Vygotsky and that of his colleagues and successors: cultural-historical activity theory (CHAT) (Van der Veer and Valsiner, 1991). In CHAT, including dialectical logic, we can find potential solutions.

Partially similar to the attempt by Ekbia (2008), but from a perspective drawing on dialectical logic and CHAT, this paper hypothesizes that, first, AI inherited the historical crisis that psychology continues to face and discusses the main feature of this reproduction of the *crisis*, i.e., its intensification (see Sections “Psychology and Its Historical Crisis: A Brief Overview” and “Artificial Intelligence Intensified the Crisis and Supported Its Denial”). Second, the paper assumes the need to overcome the empiricist tendency as a dominant direction in the field and as a main outcome of the crisis (see Sections “Artificial Intelligence Intensified the Crisis and Supported Its Denial” and “The Current Debate: The Central *Problems*”). In Section “Discussion,” a contradiction-based meaning alternative is proposed alongside a preliminary formalized model, and examples are provided. In advance, taking into consideration historical (social experience) and neurophysiological (brain characteristics as an outcome of several million years of development in terms of plasticity and connectivity) factors in the social human (or even animal) mind, we do not claim that the proposed model’s outcomes are by default a reproduction of a human-like mind. Instead, the model focuses on the process that allows abstract quality to appear *organically* from *tangible experience*.

## Psychology and Its Historical Crisis: A Brief Overview

How can psychology (and philosophy) come to the aid of AI when psychology is facing its own historical impasse, which is represented by numerous competing theoretical directions with no agreement concerning the subject matter of psychological science? Psychology has been considered to be a science facing a critical situation (Yaroshevsky, 1989) and a *problematic science* since its formation as an independent science (Teo, 2005; Dafermos, 2014). Among several attempts to investigate this crisis, what interests us most is Vygotsky’s (1997) approach. His approach is not only a tool for investigating the crisis but also a tool to overcome it (Dafermos, 2014).

Despite the fact that the *Historical Meaning of the Crisis in Psychology: A Methodological Investigation*, one of Vygotsky’s most important works, was written approximately one century ago in 1926 (Van der Veer and Valsiner, 1991), it remains under referenced and has not received sufficient attention from scholars (Goertzen, 2008). Additionally, the work “has not yet been widely discussed by philosophers and historians of sciences outside the former Soviet Union” (Hyman, 2012, p. 474), taking into consideration the fact that this work was known only to a few people before the year of its publication in 1982 (Van der Veer and Valsiner, 1991). In their debate concerning “Vygotsky’s *crisis* and its meaning today,” Rieber and Wollock declared that “history, moreover, has increased the significance of the work [Vygotsky’s work], for Vygotsky is not only a most perceptive witness to the professional crisis of his time but also a prophet of the crisis of today” (Vygotsky, 1997, p. vii). Despite the fact, that the crisis has developed (intensified), the *legacy of the crisis* has faded over time.

### The Nature of the Crisis and Its Origin

The crisis does not simply refer to the task of judging the extent to which the competitive approaches and directions are correct or not. Instead, the crisis refers to the causal question concerning how these approaches are produced and developed throughout the history of psychology. This term pertains to the objective laws and tendencies underlying those approaches. In other words, the crisis references the mind-body (subjective-objective) ontological dualism governing the epistemological and methodological development of these competing approaches alongside the growing actual practice (Van der Veer and Valsiner, 1991; Dafermos, 2014).

By serving as a source for the need for continuous methodological reform under the pressure of the practice’s principles, it was, in the final analysis, the development of applied psychology that formed the impetus and the main driving force for the crisis and that governed its future path. For Vygotsky, each approach attempted to answer the question of duality by continuing to choose duality as its starting point, even when such an approach was not conscious of that fact. By preserving the dual nature of the subjective-objective relation, attempts to synthesize the two poles were governed by eclecticism. The eclectic combination of ideas and elements from different systems resulted in a proliferation of schools and approaches, which led to an *eclectic epoch*, with a high empiricist tendency that produced a confusing mix of languages and views in the field as long as these positions defended an eclectic point of view. In other words, we stand here in front of not only one science but *many different sciences* with the name of psychology. Each science has a distinct view of the subject matter of psychological study and entails distinct facts. Therefore, it is impossible to reconcile the facts by mechanically combining them. Additionally, producing a new system cannot be completed by selecting elements from competing systems. What is required to overcome this duality is to find the *cornerstone* of psychology, its *basic cell* of analysis that can represent a *mechanism of one reaction*. In the end, the subjective is a distinct form of the objective (Van der Veer and Valsiner, 1991; Vygotsky, 1997).

### The Crisis Today and Artificial Intelligence’s Influence

Today, the crisis has become more critical and even sharper, hence threatening the coherence of psychology and watering down the foundation of scientific rationality (Leontiev, 1978; Yurevich, 2009; Al Chawk, 2011; Dafermos, 2014; Quintino-Aires, 2016). Rieber and Wollock maintained that in psychology today, “the crisis is the chaos of overdevelopment and misdirection” (Vygotsky, 1997, p. xi). However, the paradox is that a majority of modern scholars still undervalue the crisis (Dafermos, 2014), and its consequences remain undiscussed among psychologists (Augoustinos et al., 2014; Gjorgjioska and Tomicic, 2019).

This intensification of the crisis has two paths. First, there are objective sociohistorical tendencies underlying the development of science and the actions of its practitioners (Vygotsky, 1997; Rouse, 1999; Dafermos, 2014). Recently, these tendencies have influenced researchers’ thoughts and scientific practices through a high degree of syncretism, leading to a thriving fragmentation (alongside eclecticism) (Staats, 1983; Yanchar and Slife, 1997; Goertzen, 2008; Quintino-Aires, 2016). Also, these tendencies resulted in an increase in the non-paradigmatic consideration, alongside the free proliferation of theories, with a “multitude of methodological guidelines accepted at the same time” (Klochko, 2008, p. 1). This situation has led modern psychology to be *markedly heterogeneous* (Carter, 2007). Other researchers have noted the entrenchment of “realist ontology, positivist epistemology, and quantitative methods, as well as the absence of an axiological frame” (Gjorgjioska and Tomicic, 2019, p. 1), which have led to the strengthening of empiricism in the context of AI (see Sections “Artificial Intelligence Intensified the Crisis and Supported Its Denial” and “The Current Debate: The Central *Problems*”). Stam (2004) referred to the lack of commitment in psychology to the reality of the objects that it constitutes and the lack of “knowledge of theory, theory methodology, and theory needs with respect to changing from a disunified to unified science” (Staats, 1999, p. 3). Additionally, in academic and professional psychological practice, a gap exists between theory and practice, which has led to calls for an *epistemology of practice* as an alternative strategy (Polkinghorne, 1992; Fahl and Markard, 1999; Raelin, 2007; Green, 2009).

However, the topic of greater interest to us is the second path concerning the vast and rapid development of applied psychology. The actual practice establishes the tasks for science, and the levels of application within a discipline represent the progressive tendencies and objective aspects of that discipline. The level of the application contains a *germ of the future* (Vygotsky, 1997). The question of “what knowledge is and how it is acquired is a most practical question, which constantly arises in every concrete experiment, every step forward in scientific knowledge” (Mikhailov and Daglish, 1980).

Psychology is part of almost every social domain. However, what had the highest impact in the era of the fourth industrial revolution (4IR or Industry 4) is the development of AI. AI is an empirical field of *thought experiment* to artificially test and reproduce mind-related topics by using psychological knowledge. In practice, through modeling and building artifacts, AI is not only a way of *knowing* the mind and behavior but also a way of *enacting* them (Dreyfus, 1992; Crevier, 1993; Ekbia, 2008; Dennett, 2017). For Rieber and Wollock, the development of AI, as a special branch of applied psychology, was a source of increasing crisis intensity (Vygotsky, 1997, p. x).

The influence of AI on psychology is a direct outcome of the crisis. The tendency in emerging disciplines (here, in the context of AI) to become a general science is due to the absence of a general and unified coherent psychology. This tendency becomes manifest when the emerging approach in subdisciplines of psychology seeks to subordinate others, as an attempt to become a general science. An attempt for each subdiscipline to become *the psychology* in its own right, supported by the factual discoveries achieved by these approaches in their own disciplines.

The influence of AI also follows the law of the *subordination* of *ideas* among sciences and disciplines. Due to the absence of a general coherent and unified science in psychology, and due to the lack of one single accepted system and the existence of many *psychologies*, psychology has always *asked for help* by borrowing ideas from other sciences (e.g., biology, chemistry, physics) to answer questions concerning its own impasse (Vygotsky, 1997; Dafermos, 2014). For instance, the influence of quantum theory and string resonance theory on investigations of consciousness are clear examples of psychology’s tendency toward borrowing and of the law of subordination (Li, 2016; Froese and Taguchi, 2019; Hunt and Schooler, 2019).

Due to cross-domain translation between AI and psychology (see Ekbia, 2008), the development of the cybernetics approach has had a direct influence on problems arising in psychology (Leontiev, 1978). Experimental results obtained by AI research have had an apparent influence on psychology, such as the formation of computationalism and symbolic approaches, information processing theory, and the perspective of the neural network, in addition to the shift toward embodied and enactivist paradigms (see e.g., Carter, 2007; Froese, 2007; Piccinini, 2009).

Thus, a collaboration between psychology (and philosophy) and AI is already in effect. However, due to the (philosophical) crisis in psychology, the richness of facts and experience deriving from AI intensifies the crisis. In turn, due to the mutual conversation between AI and psychology, AI is affected both epistemologically (especially with respect to empiricism) and methodologically. In a word, AI inherited the crisis from psychology. In the next paragraph, we will address the intensification of the crisis.

## Artificial Intelligence Intensified the Crisis and Supported Its Denial

We assume that the intensification of the crisis in the context of AI has four main causes. One cause is the absence of a unified object of study in AI. The second cause is the empirical aspects of AI, which do not require an explicit ontological worldview. The third cause is the fragmented characteristics of tasks in AI research, which, in addition to the first and second features, increase empiricism and eclecticism. The fourth cause is the relative success of AI, which prevents critical reflection on the epistemological and methodological roots of challenges to AI, hence supporting the denial of the crisis in the field.

### The Absence of a Unified Object of Study in Artificial Intelligence

First, AI does not have its own specific object of study. In practice, AI deals with the same objects of study as psychology, such as cognitive abilities, behavior, perception, attention, language acquisition/mastery, and thinking. Additionally, one cannot detect a unified definition of the object of study among the various paradigms and models of these disciplines. For some approaches, this object is the study of the mind. For others, the object is the study of behavior or the brain (Kotseruba et al., 2020). As in psychology, the absence of a unified object of study is a symptom of the crisis (see Vygotsky, 1997). In the context of AI, this crisis is even sharper because the mission of AI research is oriented toward empirical goals intended to solve specific tasks (grasping, translation, automated driving, etc.) and not toward answering theoretical-epistemological questions as in the case of psychology as a science of the soul (*psycho-logia*) (see Ekbia, 2008). While psychology starts from an idealist position (Vygotsky, 1997), AI starts from an objective *natural* standpoint such as a position rooted in biology, chemistry, or physics. Of course, we do not neglect philosophical and psychological discussions in the context of AI, but these discussions have *external* aspects and are usually not taken seriously (Ekbia, 2008). For instance, in publications, *epistemology* stands as only one topic among approximately eighty other topics dedicated to empirical research (see e.g., Liu et al., 2018).

### The Empirical Margin and the Fragmented Tasks in Artificial Intelligence

The second reason for the intensification of the crisis in AI is the absence of a required worldview. Due to its empirical character, there is no explicit demand for AI research to produce a coherent philosophical worldview. Thus, unlike psychology and because the ideologies inherent in science cease to be hidden only when they become a worldview (Vygotsky, 1997), philosophical ideas in the context of AI can remain veiled. The concealing of philosophical ideas in AI expanded the margin of empiricism because empiricist directions do not require a guiding ontology. Therefore, by considering the fact that the empirical aspects of psychology support empiricism (Vygotsky, 1997), one can imagine how a highly empirical field, such as AI, could magnify empiricism.

The third reason is that AI is directly related to manufacturing and economic growth, domains which demand that AI become highly productive and, most importantly, specialized in specific tasks (e.g., tasks in industry, in civilian fields, etc.). The holistic aspect of the objects of study (i.e., the mind and intelligence) is lost due to this narrow focus on specific tasks, such as grasping and manipulation, attention, language processing, transportation, navigation, and object detection. Therefore, by liberating AI from an explicit philosophical and coherent worldview, the methodology of AI was also liberated from any coherent paradigm, which increased the weight of empiricism, since “science is philosophical down to its ultimate elements. It is permeated, so to speak, by methodology” (Vygotsky, 1997, p. 293). In turn, empiricism opens the door space to positivism (Mikhailov and Daglish, 1980) and “leads to the rejection of methodologically constructive principles in the creation of a system, to eclecticism…it leads to a hidden, uncritical, vague methodology” (Vygotsky, 1997, p. 300). Furthermore, in the context of AI, empiricism has two levels. One level is that of practice by researchers. The second level is the design of knowledge production and learning algorithms (e.g., reinforcement learning, analogy making, and deep learning) (see Section “The Current Debate: The Central *Problems*”).

On the other hand, the industrial/technical aspects of AI have displaced the academic classification of AI from categorization as a psychological subdiscipline to become an engineering subdiscipline. Automation schools are considered a subject for engineering and computer sciences. In addition, to attain a career in AI, the majority of researchers have an engineering background (Chella and Manzotti, 2011). Being different in terms of background and origins, researchers have various assumptions, intuitions. They have widely disparate understandings of the same concepts and practices, with a greater focus on technical aspects than on psychological aspects. Also, they are biased toward short-term outcomes based on brute-force methods (computation power and speed) at the expense of psychologically more plausible - but technically more challenging - methods (Forsythe, 2001; Ekbia, 2008).

### The Denial of the Crisis

Another factor that has intensified the crisis is its denial. In psychology, one can still find publications about the crisis (see e.g., Dafermos, 2014; Quintino-Aires, 2016); in contrast, the conceptual and terminological contents of AI research have not yet reached the level of *crisis.* What is present in this context are the concepts of *impasse* (only in a few pieces of the literature) and *challenge*.

By searching the *Google Scholar* and *Scopus* search engines for the words *crisis* and *artificial intelligence*, one cannot find pieces of the literature dedicated explicitly to the root of the crisis. Furthermore, when the word *crisis* is used, what is meant by that term are its outcomes (symptoms) such as the absence of a unified object of study, different competing directions, and the reform of methodology in the context of actual and objective tasks (e.g., Fuernsinn and Meyer, 1970; Tienson, 1988; Swann, 1992; Stojanov, 2001; Lindblom and Ziemke, 2003; Chella and Manzotti, 2011; Kaur, 2012; Hála, 2014; He et al., 2017; Hernández-Espinosa et al., 2017; Hutson, 2018; Kotseruba et al., 2020). However, a draft by Smith (2019) noted the fact that AI is undergoing a crisis. He suggested that AI requires paradigm reform. The reform aims to allow the agent to intrinsically and meaningfully perceive the content and substance of sensory input by allowing the introduction of knowledge from the sensory streams. These streams represent the semantics and functionality of the relationships, not only as a result of the external shapes of the phenomena in question. However, the draft only depicted general highlights and did not investigate the epistemological roots of the crisis.

Another important cause of the concealment of the crisis is the relative success of AI in recent years (see e.g., Sarker, 2021). The share of AI in the market has expanded, reaching approximately 100 billion USD at present, with the annual growth rate of this segment of the market estimated to be 40.2% from 2021 to 2028 (Grand View Research, 2021). This relative success provided methodological legitimacy for several prevailing models in AI and protected them from questions. Regarding the number of publications, Liu et al. (2018) found that between 2000 and 2015, the number of published papers in only nine key journals and twelve key conferences was approximately fifty-nine thousand (59,000) papers receiving approximately a million and a half (1.5) citations. Moreover, in Liu et al.’s (2018) investigation, *epistemology* as a research topic stands as only one of approximately eighty other topics (genetics, astronomy, finance and microeconomics, pixels, databases, quantum mechanics, developmental and cognitive psychology, etc.) and is a relatively small area of interest. These facts are straightforward and quantitative examples of the high proliferation and increased weight of empiricism in the context of AI and of the denial of the crisis through the reproduction of the same positions with new forms and under new labels that swiftly but gradually merge with one of the poles (rationalism and naturalism) (Ekbia, 2008).

What prevents any revision of the roots of the crisis is the tension and the gap between *scientific* (theoretical) and *engineering* (technical-empirical) *practice* in the field. The more epistemological tension there is, the more approaches and publications there are. On the other hand, the exaggerated success of AI is partly derived from the researchers’ intention to obtain and maintain the large amounts of funding gained by the *big science* (AI) (Ekbia, 2008), such that many researchers have “made misleading claims of success in some areas” (Palij, 2009, p. 3). These conditions have narrowed the scope of critical reflection in the context of AI in the sense of evaluating the backgrounds (principles, assumptions, biases) that guide the creation of theories, models, and technical systems. Additionally, researchers have tended to disregard the shortcomings of their research to magnify their own achievements. Researchers have been influenced by the commercial and promotional aspects of AI, and hence have reflected a tendency to communicate certain beliefs concerning favorable links between AI and the mainstream social order (Kling and Iacono, 1990; Rouse, 1999; Ekbia, 2008).

### Eclecticism, Compromising, and Proliferation

For these reasons, researchers have been compelled to become eclectic and to “equally” accept psychological ideas in an acute version of the eclecticism of modern psychology (see Klochko, 2008). For these researchers, all psychological directions have become true, even when these ideas contrast and the *assumptions* of these ideas are “often taken for granted in technical work, and that might therefore be at the root of problems” (Ekbia, 2008, p. 15). Eclecticism has furthered the need to conflate various contrasting methodologies and has increased the proliferation of models and designs. Also, it strengthened the tendency toward the selective adoption of ideas *via* an additive, mechanistic method, which represents a shift away from the holistic and coherent structure to which these ideas belong. This situation can clearly be seen in Ebkia’s assumption that no single (philosophical, psychological, informatic) approach and model can explain cognition by itself! In addition to the vague, ambiguous, and imprecise translation of psychological knowledge and discourse into the context of AI as a form of *cross-domain allusion* (Agre, 1997), the noted situation has propagated a non-critical attitude, hence interfering with technical practice (Varela et al., 2017). It resulted in muddled and misleading claims, rival research projects working on the same topics, and unjustified redundancy (Ekbia, 2008).

Regarding the tendency toward compromise, some pieces of the literature have considered Vygotsky and Piaget to be equivalent (see e.g., Stojanov, 2001; Maia et al., 2015), neglecting their radical contradictions (see Vygotsky, 1986, p. 96). Additionally, in contrast to Vygotsky, other researchers have maintained that concepts, meaning formation, and language acquisition are, for Vygotsky, based on the direct associations among the components of experience (e.g., Billard et al., 1998; Billard and Dautenhahn, 1999; Mirolli and Parisi, 2011; Emel’yanov et al., 2016). In some models, the role of the Vygotskian socio-historical context in mental development has usually been reduced to direct external interaction among social actors (see e.g., Lindblom and Ziemke, 2003). Vygotsky has served as an example here because his proposed framework contains a clear methodology and makes no ambiguous assumptions.

This tendency toward a misinterpretation and mixture of contrasting ideas reflects the lack of proper theorizing as a symptom of the crisis (Dafermos, 2014), while practitioners move freely between different notions and conceptions in AI. This situation may be a result of the fact that AI is still finding its theoretical foundation (Sharkey and Ziemke, 2000). However, a simple combination of conflicting theoretical directions, as epistemic practice, by no means provides us with a *new system* (Vygotsky, 1997).

Furthermore, regarding the proliferation of models, since the mid-1950s, the number of ‘cognitive architectures’ [e.g., Learning *Intelligent* Distribution Agent (LIDA), Adaptive Control of Thought–Rational (ACT-R), State, Operator, and Result (SOAR), Connectionist Learning with Adaptive Rule Induction On-line (CLARION), etc.] has increased to approximately three hundred. Furthermore, dozens of models are currently being adopted, in addition to thousands of models for each task (navigation, manipulation, etc.), which is reflected in a large number of publications regarding each topic (Liu et al., 2018; Kotseruba et al., 2020). In addition, all these models and architectures pertain to reproductions of the same mental processes.

### Other Outcomes of the Crisis’ Intensification

#### Artificial Intelligence as a Duplication of Psychology

Based on the increased weight given to empiricism and the lack of theorization, AI is not only reinventing the wheel; it is also becoming a sort of duplication of psychology. In terms of the logical and historical production of thoughts (see Vygotsky, 1997), we can see a tendency toward analogy and parallelism between both fields (see e.g., Balkenius, 1995; Stojanov, 2001). For Stojanov (2001), AI research in the mid-1980s was similar to psychology in the 1930s when AI shifted from cognitivism (which had governed the field since the 1950s) toward a developmental direction. This shift represented the transition from a rationalist position toward an objective standpoint (see Vygotsky, 1997). However, as noted previously, this repetition also took the form of inflation and enlargement. Due to AI’s *empirical elasticity*, each psychological approach translated to the context of AI can appear in multiple versions. For example, as a symptom of proliferation in the form of hybrid approaches, to overcome the challenges of reinforcement learning (RL), deep reinforcement learning (DRL) emerged through the addition of certain elements (e.g., the introduction of cognitivist elements such as the hierarchy architecture and through an increase in the complexity of the policy and the associated rewards) without affecting the epistemic principles of RL (Amarjyoti, 2017) (see Section “The Current Debate: The Central *Problems*”). Another example of empirical elasticity is the introduction of recurrent neural networks to solve the lack of a time concept in the context of an artificial neural network (ANN) by adding complexity to the layers to create a memory for the system. Such changes are only carried out *at the project level* and not at the *programmatic level*, so they still share the same *foundational programmatic flaws and problems* (Bickhard and Terveen, 1996; Ekbia, 2008).

The intensification of the crisis has resulted in a faster elaboration of the crisis than in the case of psychology. It took nearly four decades for AI to reach the epistemological and methodological impasse that required several centuries for psychology to reach, thus revealing the *short, tumultuous*, and *intriguing* history of AI (Crevier, 1993; Ekbia, 2008). Furthermore, each newly taken direction required a shorter period to confront its epistemological and methodological challenges compared to the previous direction. The rationalist (i.e., cognitivist, encodigism, top-down) direction dominated for approximately three decades, from the early 1950s until the 1980s - that is the date of the first impasse (e.g., Dreyfus, 1981) - while the naturalist (i.e., emergent, bottom-up) direction needed only one decade (the 1990s) to confront its difficulties – that is the context of the second impasse (e.g., Bickhard and Terveen, 1996). It is considered a second impasse because the widely adopted interactivist and emergent positions (*embodied*, *enactivist*, *behavior-based*, and *situated*) “are at least as selective as the older reasoning-based approaches that they criticized, though in different ways” (Sloman, 2014, p. 8).

Since then, a debate between the two directions has existed, giving the crisis its current appearance. It is worth noting that each direction is not present independently in various approaches. Instead, both the rationalist and the naturalist direction coexist and define the internal structure of each approach, as in the case of psychology (see Vygotsky, 1997).

#### The Increased Weight of Naturalist Direction

However, the main aspect of this situation is the increased weight given to the naturalist position at the expense of directions derived from the philosophy of mind. Apart from a purely idealist position rooted in symbolism that does not have any epistemic access to the world, i.e., the problem of the *mind in a vacuum* (Bickhard and Terveen, 1996; Sharkey and Ziemke, 2000), naturalist approaches have introduced materialist aspects. Aspects such as the role of brain activities (in the case of connectionism and its realization in the form of artificial neural networks) (Carter, 2007) and the role of mechanistic and living bodies (in the case of embodiment) (Sharkey and Ziemke, 2000; Ziemke et al., 2008). However, due to the subjective-objective gap, progress toward the naturalist position has been realized by ignoring the subjective and mental qualities, e.g., ignoring the role of symbols and representations, or by abandoning the goal-satisfaction principle, resulting in a form of physicalist reductionism. Connectionism is a version of neural reductionism, while the mainstream conception of embodiment and situatedness is an anti-mentalist version of physical and biological reductionism and even of eliminativism that employs the formula of *life* = *cognition*. For instance, the term *emerge* has been abandoned by some proponents of embodiment and situatedness and replaced by the term *integration* (Horgan and Tienson, 1991; Ekbia, 2008). Additionally, embodied approaches have only extended connectionist approaches into the bodily domain. What we have now, instead of brain-based neural reductionism, is bodily sensorimotor reductionism. Nevertheless, the mediation among new elements has followed the same associationist, connectionist, and statistical approaches. This situation is the case for the hybrid mechanical combination of symbolism and robotics (the *grounding problem*) that has been undertaken using numerous methods: existential programming, reinforcement learning, genetic algorithms, and deep learning (Sharkey and Ziemke, 2000; Sloman, 2014).

Furthermore, in confronting these forms of reductionism, recent invitations have even proposed a *radicalized* biologism and physicalism, e.g., the Meta-Morphogenesis Project’s proposal concerning the biological evolution of information processing and biology-based robotics (e.g., metabolism-based cognition) (Sharkey and Ziemke, 2000; Ziemke et al., 2012; Sloman, 2014; Ziemke, 2016). Another outcome of the *failure* of the first version of the embodiment position is the ontological rejection of emergence and causality. Some researchers have replaced the term *emergence* with *integration* (Ekbia, 2008) or produced hybrid versions combining both embodiment and mentalism, e.g., enactivism, as an attempt to radicalize embodiment’s *living body via* the phenomenological insertion of *the first-person* point of view *via* the so-called *subjective living body* (Froese, 2007; Varela et al., 2017). However, it is “not yet clear how a concern with subjective experience could provide us a way to” move forward (Froese, 2007, p. 11).

These views have attempted to *solve* the problem of differentiating between the mental and the physical by neglecting the problem in the first place as a result of the influence of panpsychism (as an assumed middle ground between materialism and dualism), e.g., anti-emergent panpsychism. For instance, the argument that the combination of components provides phenomenological experience was influenced by panprotopsychism. However, even in panpsychism, as a symptom of the crisis in philosophy, the problem of dualism is still preserved in the *combination problem* (see e.g., Bruntrup and Jaskolla, 2016; Benovsky, 2018). Additionally, even though panpsychism has gained gradual acceptance in science, especially in neuroscience (Koch, 2012, 2019), it is considered to be a metaphysical version of the depsychologization of consciousness by consigning consciousness to a metaphysical limbo beyond the reach of science (Goff, 2009; Frankish, 2021). These attacks, counterattacks, and the mechanistic combination (attachment) of both tendencies have defined the overall path of AI research (Ekbia, 2008).

We can see that in mainstream *naturalistic* tendencies, ontological dualism is preserved, either by reducing the mind to something purely physical (biological) by implicitly considering the subjective to be non-material or by *injecting* the subjective into the material world, as in the case of hybrid models. This situation appeared in the same way in the context of psychology a century ago. Overall, along with the development of the field, the naturalist position in AI has increased in a manner similar to psychology (see Vygotsky, 1997).

Next, we will introduce the crucial central problems as identified by the body of knowledge in the field, which are rooted in the empiricist epistemology derived from the gap resulting from ontological duality.

## The Current Debate: The Central *Problems*

During its *short* and *tumultuous* history, AI research has elaborated central *problems* and key propositions. Our attempt does not exhaust all of these topics but focuses on those shared among different frameworks, approaches, and scholars. By so doing, one can identify the requirements necessary to understand the semantics, interpreting and mattering (making meaning and sense for the *user of the representation*), active engagement and autonomy, a reflection of reality, abstraction, and generalization that are crucial for learning and adaptability. However, below, we present the drawbacks of mainstream approaches to these *problems* and quests.

### Encodigism, Symbolism, and Connectionism

Starting from the clear idealist positions of encodigism and symbolism, the body is detached from its context in a closed circular system of rules-based syntaxes and the data structures of a world model, in which there is no relation to semantics and meanings (e.g., Searle’s, 1980 Chinese Room). The mind is static, has no epistemic access to the world, and cannot be updated, i.e., the *frame problem*. The mind is *incoherent* because no new elements *emerge* outside those that are pre-encoded. Additionally, the mind is *circular* because its representations are interpreted by other representations. Therefore, *causality* is out of reach. Cognitivism considers mental functions to be explicit and intelligence to be an exhausting *search* process. Furthermore, these approaches have failed to engage with the problems of *functionality*, *mattering*, and *interpretation* as crucial aspects of conscious existence. In addition, these predesigned models are inconsistent with the dynamic character, continuity, and complexity of reality, especially when it is impossible to plan *in advance* for all potential states and situations. Overall, symbolism is philosophically idealist and leads to skepticism (Bickhard and Terveen, 1996; Ekbia, 2008; Dennett, 2017). “The rationalist tradition had finally been put to an empirical test, and it had failed” (Dreyfus and Dreyfus, 1991, p. 45).

On the other hand, connectionism introduces the subsymbolic principle of representations formed by the physical states and neural activities of the brain (*via* the activation and weighting of nodes and connections). Here, semantics are derived from the functional roles of states as the mediator between inputs, outputs, and other states. Connectionism represents depersonalization and desubjectivization and has a *mysterious quality*. The agent’s active role is still missing in the formation of *generalizations* because generalization is different from the process of filtering the categories of the world through networks. Additionally, unlike the human abilities to reperceive and reconfigure, learning by connectionist networks is *inflexible* due to a lack of any level of *abstraction*. This situation leads us to the shortcoming in the grasping of *meaning* that appears clearly in connectionist models of language learning, which focus only on the external features of phenomena, in addition to the problems of binding and high dimensionality (Bickhard and Terveen, 1996; Ekbia, 2008).

### Hybrid Models, Learning Algorithms, and Embodiment

Furthermore, even for learning algorithms in the hybrid *adaptive* and *emergent* models (in training artificial neural networks), numerous *problems* exist, e.g., a long training period, the inability to engage in abstract learning and generalizing skills among contexts, difficulties in synthesizing (fusing) the elements, concept formation, the emergence of symbols and meanings, the grounding problem, and functional reflection (e.g., Ziemke and Sharkey’s, 2001; Guerin, 2008; Stojanov, 2009; Kober et al., 2013; Borghi and Cangelosi, 2014, Taniguchi et al., 2018; Froese and Taguchi, 2019).

We maintain that these problems are the result of the empiricist understanding of knowledge, which stems from the gap produced by ontological duality. In summary, so-called *emergent* systems, in their mainstream version, are mechanistic, associationist, statistical, and purely sensualist.

For instance, reinforcement learning (RL) in the context of robot learning represents the trial-and-error methods of behaviorism and “attempts to explain the development … from the viewpoint of the mechanistic principle of the accidental combination of heterogeneous elementary reactions” (Vygotsky, 1997, p. 201). This technique excludes the process of thinking and reduces development to a stimulus-response relationship (Vygotsky, 1997). Furthermore, “the informational function of reward and punishment is limited because there is no understanding of the stimulus-response relationship” (Bedny and Karwowski, 2006, p. 350). By representing positivism that is *devoid of an active person*, behaviorism cannot explain the problem of mattering and interpretability because there is no reference entity for the process of meaning formation. By reducing intelligence to merely sensor-actuator mechanistic behavior through a process of blind trial-and-error, RL cannot understand the higher complex mental activity that results in long-term learning. For RL, the active perception and semantics remain open problems (Vygotsky, 1997; Cruse et al., 2000; Bedny and Karwowski, 2006; Carter, 2007; Kober et al., 2013). For Vygotsky:

*“The description ‘this animal is running away from some danger,’ however insufficient it may be, is yet a thousand times more characteristic for the animal’s behavior than a formula giving us the movements of all its legs with their varying speeds, the curves of breath, pulse, and so forth” (Vygotsky, 1997, p. 277).*

Additionally, by recording the successive concurrence pattern of action-context results, the process of making analogies neglects reflective abstraction (as a crucial process in knowledge production) and accounts primarily for empirical abstraction (regarding external features). In analogy making, the learning process is based on similarity and familiarity that is derived from constructivism in psychology (the Piagetian position) (see Drescher, 1986, 2003). In addition, constructivism does not provide a “mathematical” model for integrating the elements of experience or concerning how to shift from one stage of development to the next, and it is not clear how these different levels of abstraction operate (Ekbia, 2008; Stojanov, 2009; Kelley and Cassenti, 2011). By relying on “temporal sequences and by the application of a mathematically conceived formula of the functional interdependence of phenomena,” Piaget replaced the “explanation of phenomena in terms of cause and effect by a genetic analysis” (Vygotsky, 1986, p. 96).

Furthermore, following connectionism, deep learning tries to mimic the human brain and needs an enormous number of datasets, since it depends only on the number of associations among elements to form a *pattern of data* (see e.g., Kotseruba et al., 2020; Sahu and Dash, 2021). Additional examples of the noted aspects can be found in other machine learning algorithms (see e.g., Dash et al., 2021; Sarker, 2021). For these bodily reductionist forms of embodiment, the organism is merely a puppet controlled by an environmental puppeteer (Sharkey and Ziemke, 2000), which leaves no room for subjectivity (Dennett, 2017).

Based on the inadequacy of weak embodiment, a call for *strong embodiment* appeared to allow meanings to emerge, a development which was influenced by *Uexküllian embodiment* and the proposition of integrating an organism’s components into a subjective, purposeful whole (Sharkey and Ziemke, 2000). For Uexküll, the organism-environment interaction is always functional. The environment’s objects are the carriers of meanings, and the organism is the analyzer of meanings (Uexküll, 1982). Since there is no formalized model in *Uexküllian embodiment* for the emergence of meaning, some researchers have drawn on Uexküll’s notion of a subjective, purposeful whole to *inject* the subjective externally into natural existence, e.g., *enactivism* (Sharkey and Ziemke, 2000; Varela et al., 2017).

### The Axis of Argumentation: Qualitative vs. Quantitative

In summary, the axis of argumentation found in the literature is based on the project of making sense from the experience semantics. From the *viewpoint* of an interpreter, it is required to develop abstract levels of knowledge that can reflect the complexity of reality and allow for generalizations. These points are crucial to other *problems* in the field.

Overall, in regard to *information* representation as an *engineering* question, and despite the theoretical differences among mainstream frameworks, the technical realization follows a formal and quantitative/statistical methodology, e.g., Markovian and Bayesian (see Du and Swamy, 2013). Also, the mainstream approaches follow a mechanistic, connectionist, and associationist path, even when the notion of socialization is introduced (e.g., in the context of social actors or swarm intelligence). This fact is a result of the mainstream empiricist direction: the purpose of knowledge is to record external features of the phenomenon (e.g., shape, color, speed, cooccurrence, level of drivers) as well as the temporal and spatial relationships among phenomena (e.g., the weighting of connections and nodes, temporal occurrence, accumulation of costs and rewards). Doing so by solely exploring the content of the sense organs with no theory concerning methods of grasping meaningful events under the formula that “all we needed was more of the same” (Dennett, 2017, p. 86) (see e.g., Mikhailov and Daglish, 1980; Vygotsky, 1997; Cruse et al., 2000; Dafermos, 2014). Quantitative measurements are overestimated due to the separation between the technical function of science and theoretical thinking (Dafermos, 2014). Quoting from Münsterberg, Vygotsky noted that the majority of researchers “write out the last decimal point and put great care and precision in answering a question that is stated fundamentally incorrectly” (Vygotsky, 1997, p. 258). Statistical analysis is a limitation derived from empirical generalization based on the notion of simply classifying common characteristics among static objects (Ilyenkov, 2009). Overall, the quantitative tendency is supported by the *brute force* of speed and computing power (Ekbia, 2008).

Additionally, the subject/person is considered to be the summation of the parts (e.g., in the case of functionalism), thus adopting a subpersonal position or indicating a person-vacuum (a positivist *mindless-body*) standpoint; hence, this viewpoint does not constitute a purposeful whole (Haselager, 2005; Haselager and Gonzalez, 2007; Dennett, 2017).

Therefore, one should ask: are current models *emergent*? To be emergent means that new *qualities* and *sorts* of things appear in existence. These things did not exist previously, and now they do exist. Such things differ in terms of quality from things that originated in contexts prior to those in which the new things emerged, e.g., in the transitions from non-representational to representational, physical to mental, objective to subjective, or simple to complex. To be emergent means to include the principle of causality (see e.g., Bickhard and Terveen, 1996; Cruse et al., 2000; Ekbia, 2008; Estrada, 2014). Thus, so far, the mainstream models are not emergent.

In the next section, we discuss how, in contrast to formal and mechanistic approaches, dialectical logic and CHAT may provide answers to these problems.

## Discussion

### CHAT in Brief

Unlike mainstream directions, the reflection of reality and knowledge/thought production cannot be deduced statistically from sense organs alone. Additionally, qualitative phenomena cannot be reduced to quantitative phenomena (Leontiev, 1978; Mikhailov and Daglish, 1980; Gribanov, 1981; Vygotsky, 1986, 1997). The reflected picture of the world is “accumulated not only directly at the sensory level but also higher cognitive levels…In other words, the “operator” of perception is not only simply the previously accumulated associations of sensation” (Leontiev, 1978, p. 41). Additionally, brain mechanisms are not the explanatory basis for developmental psychological processes and higher mental functions (Luria, 1966, 1976; Leontiev, 1978; Vygotsky, 1997).

In this study, we have the *philosophy of activity* instead of the *philosophy of mind* or the philosophy of *biological body/brain* or *pure physical behavior*. CHAT investigates the mental as a sociohistorical phenomenon both ontogenetically and phylogenetically. The mind (e.g., mental functions, personality, self, identity, intentions, consciousness, and the unconscious) is the outcome of *social activity as a system*. Additionally, as for other material phenomena (nature and society), the activity system and its outcome (the mind) are governed by dialectical laws. Real activity (and not phenomenological experience) is the starting level for investigating mind activity (the second level, i.e., the psychological level). The third level (the neurophysiological brain) is only the plain on which the first two are represented. To adapt, the active organism is driven causally by needs and desires. By seeking satisfaction, the activity confronts, as Hegel noted, the resistance of the environment and that of the agent’s body itself, i.e., the force of nature and its material, including ready-made sociohistorical relationships and social actors, tools, objects of desire, culture, language and symbols (Mikhailov and Daglish, 1980; Marx and Engels, 1996, 1997, 1998). It is “in this process, by acting on external nature and changing it, he [the human being] at the same time also changes his own nature and acts upon it” (Vygotsky, 1997, p. 87). The activity is “a molar, not an additive unit of the life of the physical, material subject” (Leontiev, 1978, p. 50). Therefore, meaning-based (psychic) reflection, as a *twofold transition*, “emphasizes the constant flow of activity as the source of mind and self” (Stetsenko and Arievitch, 2004, p. 484).

First, subjectivity must include the reflected practical goal-oriented activity (the practical role of the organism) in the activity once again, hence leading to the emergence of the active components of activity (mental activity and actions). The second transition occurs when these active components become the object of another reflection, i.e., self-reflection. For example, the action of the organism pushing an object becomes a mental component of *an active actor* (I am doing). This situation is different from the recurrence and mirrored reflection found in mainstream studies. Here, like all other features, self-recurrence gains qualitative content by introducing new qualities to the system. For instance, the formation of the *self* introduces new components, relations, and laws. The self is the “embodiment of a meaningful project…that reflects and also organizes the most significant aspects of one’s life” (Stetsenko and Arievitch, 2004). The evaluative role of the *self*, regarding the *signs* of the experience, lies in the core of *interpretability*. The *self* becomes a constraint on activity and a source of new needs, desires, and shapes of the mental structure.

The emergence of subjectivity, including the self, is a material process that originates entirely in the flow of activity as a process in movement (Stetsenko and Arievitch, 2004). This fact has been verified by schizophrenia studies, while the disturbance of the flow of activity impairs mental structures and processes, e.g., self-regulation (Warner and Mandiberg, 2003; Marwaha and Johnson, 2004; Scherder et al., 2010; Meijers et al., 2015; Semenova, 2020). Additionally, the role of movement as a source of subjectivity has been noted in psychotherapy through the self-moving dynamic unity of body-mind (see e.g., Dobrowolski and Pezdek, 2021). However, unlike CHAT, the self-pattern theory and the standpoint focused on bodily actions and movements both represent enactivist/phenomenological versions of expressing the role of the flow of activity in forming the self, especially by considering the self to be a *narrative;* hence, this model represents a direct associationist-mechanistic and radical empiricist position (see e.g., Dennett, 1993; Gallagher, 2013; Popova and Ra̧czaszek-Leonardi, 2020).

In addition to subjectivity, CHAT includes central components that AI systems must have, e.g., semantics and intrinsic meanings, interpretation, and needs and goals (e.g., internal drivers in the case of the embodiment, network constraints in the case of connectionism, constraints pertaining to goals and rules in the case of formal symbolism) (see e.g., Bickhard and Terveen, 1996; Haugeland, 1997; Ekbia, 2008; Dennett, 2017). However, for CHAT, these components play a causal and qualitative role by serving as part of the dialectical framework of contradiction formation (see Section “Meanings and Contradictions: A Representation of the Dialectical Process”).

The purpose of this discussion is not to explore CHAT exhaustively, but what does interest us is the way in which CHAT engages with the *problems* at hand by focusing on the ways in which meanings emerge as the core of reflection. Following the method of *analysis by units*, unlike the positivist method of *analysis by the element*, meaning is the basic unit that entails other *problems* in intelligent complex systems. These problems include abstraction, generalization, interpretation, self-reflection, concept formation and language acquisition, emotions, and intellect unification (Vygotsky, 1997). Meaning serves the purpose of including a subjective-meaningful entity in the context of experience (Froese, 2007).

### Reflection, Meanings, the Fabric of the Mind, Self, and Subjectivity

For Vygotsky, development is an adaptive process, which is realized by confronting (facing and overcoming) perturbation in the context of the activity. The outcome is the emergence of meanings representing the qualitative content and internal substance of the agent’s experience. These meanings mediate the agent-environment interaction - from the most straightforward motor action to higher mental functions - to produce new meanings that mutually constitute the dynamic system of meanings (DSM). DSM is the context in which the components of experience are integrated and synthesized, forming the structure of the human mind, including action, needs and desires, goals, external sensory inputs, and the internal outcomes of the experience (emotions and affection) (Leontiev, 1978; Vygotsky, 1986; Babaeva et al., 2013).

From a similar (but away from CHAT) position, Menant (2011, 2020) maintained that, by seeking satisfaction, *internal constraints* (needs) generate useful meanings by receiving information from the environment (the agent in action). These meanings ground the agent in the environment through directed actions taken to modify the environment, resulting in the functional production of knowledge and allowing perception to be realized (Menant, 2015). Additionally, for Frankl (1992, 2014), adaptation is a process of meaning generation through the meaning generator system (MGS). However, due to Frankl’s existential position, his methodology follows a mentalistic and phenomenological path and does not intend to engage in any structural or causal investigation. Regarding this *historically void* position, we quote: “But no man can tell another what is this purpose (the purpose of living and the meaning of life); Each must find out for himself” (Frankl, 1992, p. 9). Even Frankl admits that conflicts, predicaments, and suffering can be transformed into a human achievement. However, for him, it seems that suffering is not the rule but merely the exception (see e.g., Frankl, 1992, p. 117 and 140).

For our topic, i.e., mind formation, *meaning* resembles the unit that is “the part of an organism that retains all the essential characteristic of the whole organism” (Dafermos, 2014).

The formation of personal meanings is “a special form of psychological reflection” (Babaeva et al., 2013, p. 12).

The role of meanings in thinking (e.g., the regulation of thinking) is decisive in that it realizes the functional reflection of reality (the relationships among components of experience), hence forming the fabric of consciousness (psychic images or concepts). Furthermore:

*“Personal meanings formation was defined as the procedural and structural development of personal meanings in the course of human activity, which integrated the processes of creation and the functioning of the cognitive structures (images, concepts, and knowledge), goals, and the emotional and motivational components of thinking” (Babaeva et al., 2013, p. 12).*

In meanings, intellect and affect unite through the “dynamic system of meanings” (DSM) (Leontiev, 1978; Vygotsky, 1986, 1997; Bedny and Karwowski, 2006; Babaeva et al., 2013). That unity “’of the affective and intellectual processes,’ has been understood to mean the unity of the functional development of the cognitive and personal regulation of thinking” (Babaeva et al., 2013, p. 12). For CHAT, emotions are considered in terms of *intellectual emotions* and not merely as somatic components. These emotions carry out a *delicate* form of regulation:

*“as they influence the structures of cognitive activity… Emotional experience precedes the objectification of gnostic contradiction and the setting of the goal of cognitive activity, it initiates and directs the search for the logical structure of a contradiction. This phenomenon is called the ‘emotional detection of a problem” (Babaeva et al., 2013, p. 8).*

Furthermore, Luria (1966, 1976) held that the brain is a unified system of functions and not merely a basis for mirrored reflection (directly recording sensory-motor inputs). For Luria, the psychological processes are the basis of understanding the cortical structures and the dynamic system of the brain (not vice versa). The working brain is a system of interconnected analyzers that integrate inputs from direct sensorimotor experience toward higher control functions by way of analyzers. These analyzers are interconnected and support each other through a hierarchical structure according to which the higher analyzers (the overlapping zones of analyzers representing cortical interconnections) include the lower ones. For example, peripheral receptors connected directly to the sense organs are not directly analyzed but are analyzed only through several layers of analyzers. Therefore, in neurophysiological language, these interconnected analyzers represent the brain version of the dynamic system of meaning (DSM).

Meaning is not the destination of an intelligent system. It is the starting point. Meaning is the ghost hovering above the field and the *barrier* that the field needs to overcome to realize crucial abilities: language and semantics, symbolization, interaction, complexity, intelligence, learning, etc. (Cruse et al., 2000; Carter, 2007; Ekbia, 2008; Dennett, 2017). Thus, an artificial model must grasp the process of meaning formation by adopting dialectical logic.

### Meanings and Contradictions: A Representation of the Dialectical Process

The mainstream formal logic-based and mechanistic tendencies result in a flat world limited to horizontal expansion in contrast to a self-developing system. Instead, for dialectics, the internal contradictions of any system are the source and internal motive for the development of that system, including the mental (Kosok, 1966; Tikhomirov, 1988; Klochko, 2008). For Hegel, it is only due to contradictions that something can move, become active, and have its drive (Miller, 1977).

From Riegel, we quote the following:

*“From a dialectical perspective, change and development are a result of contradictions between events occurring in different progressions, such as biological, psychological, or cultural-sociological progressions. The resolutions of these contradictions, or crises, provide the basis for further development - both positive or negative – of the individual…” (Riegel, 1979, p. x).*

In this regard, the meanings are the outcome of the process “where a person is solving a problem and the detection of contradictions in the objective properties of the task,” and “the attempt to adjust the contradictory properties of the object leads to different representations of the primary operational meaning of the solution attempt” (Babaeva et al., 2013, p. 13).

In this sense, the adaptive system is more than *homeostatic* or *autopoietic*. Such a system is not only a matter of “maintaining parameters which are crucial for system’s preservation within the tolerable limits” (Klochko, 2008, p. 31). In contrast to maintaining constant self-organization and equilibrium (Maturana and Varela, 1987), the developmental system is self-developing, a *transcendental* system exhibiting a continuous increase in complexity and organization (Klochko, 2008). It is a matter of *being through becoming* (Kosok, 1976).

Moreover, power is gained “only by looking the negative in the face and tarrying with it. This tarrying with the negative is the magical power that converts it into being” (Miller, 1977, p. 19). For Vygotsky, the “negative” experience is the productive side of any crisis (Vygotsky, 1997). Furthermore, dialectical negation is held to govern subjective judgments (Smith et al., 1995).

Including the role of *negative* experience is in line with the role of error in *genuine learning* that is noted in AI (Bickhard and Terveen, 1996). Unlike the case of mainstream learning frameworks (e.g., encodigism, reinforcement learning), these errors have to be self-generated and not preprogrammed and must have a meaning in order to be *wrong from the perspective of* the system itself; otherwise, all inputs (error, success) become equal in terms of quality. To learn is not to avoid error but to undergo a sort of *system variation* when encountering the error and to generate new *error criteria and signals* (Bickhard and Terveen, 1996, p. 58). Moreover, *negative* experience is an abstraction of the *pain argument*, which is considered crucial for the system to experience a phenomenal state (see e.g., Carter, 2007; Dennett, 2017).

Cultural-historical activity theory (CHAT) is not new in the context of AI. However, no studies have presented the crucial role of contradiction (see e.g., Lindblom and Ziemke, 2003; Kofod-Petersen and Cassens, 2006; O’Leary, 2008; Mirolli and Parisi, 2011; Huang and Mutlu, 2012; Suchan and Bhatt, 2012; Dhuieb et al., 2015; Maia et al., 2015; Emel’yanov et al., 2016; Gonçalves et al., 2017; Tramonte et al., 2019). It is paradoxical to instrumentally accept CHAT “without serious reflection on the complex formation process of its theoretical background” (Dafermos, 2014, p. 148). Tang et al. (2020) stressed Vygotskian meaning production in the context of learning processes (including transferring learned skills, abstraction, and problem-solving) as a dialectical interaction. However, the meaning was only one part of their general discussion, and they did not intend to explore the structure of meaning or how a robot can functionally reflect reality. The robot’s role was to only aid the human specialist in the development of meanings.

Furthermore, according to Hegel, a contradiction is the unification of opposites (Miller, 1977). Kosok (1966), for one, attempted to formalize (algorithmically) dialectic logic (see Riegel, 1976; Counet, 2012) by introducing the structures of contradiction formation and dialectic interaction in the social sciences.

For Kosok (1966), contradiction is the moment of negation, when an entity *B* (the antithesis) negates an entity *A* (the thesis). Unlike formal *standard negation*, which considers *B* to be distinct from *A*, *B* is instead the *positive absence* of *A* (presence as a lack), which is represented as *¬A* (*not A*, or -*A*). The negation relationship (¬) is crucial for contradiction formation. As the representation of a continuous flow of movement (see Section “CHAT in Brief”), the dialectic process in any system is a continuous, exponential self-reflective-expansive system, in accordance with the fundamentally recursive Formula (1) (Kosok, 1966, 1976).


(1) $$\left(R\right)e^{n}=e^{n+1}$$

where *R* is the process of reflection, and *e* is the reflected entity. The first step is the negation of *e*. The outcome is the assertion of *e* (+*e*) and the absence of *e* (−*e*, not *e*, or *¬e*). In addition, the outcome of *R*, i.e., *e*^*n*+1^, is embodied in the initial elements of the coupling, i.e., *e* (+*e*, and −*e*) (Kosok, 1976).

The process of negation uncovers/abstracts the substance of the phenomenon and allows for the conception of its internal content (Davydov, 1990). Abstraction as a “content-related” process is meant to isolate and “mentally retains the specific nature of the real relationship of things that determines the formation and integrity of assorted phenomena,” as an analysis of the “function and role of a certain relationship within a certain system” (Davydov, 1990, p. 138). Later, to generalize is to employ what is already abstracted.

**FIGURE 1:**
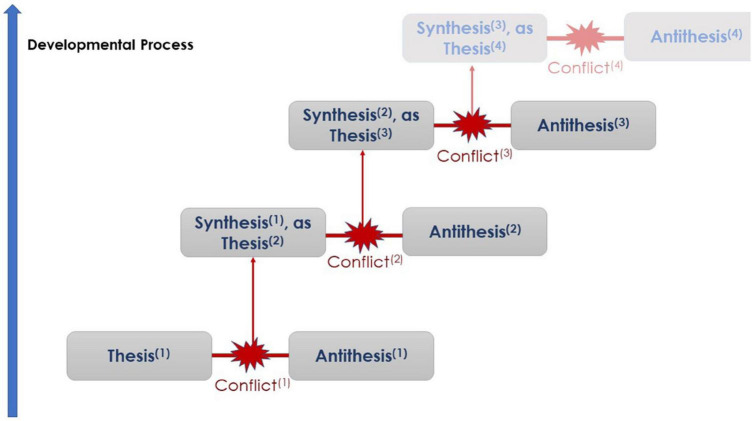
The basic form of the Hegelian triadic structure.

By grasping the substance of the phenomenon (the coupling of contradictory entities) through a continuous process of negation (and later the negation of the negation), new qualities appear, i.e., *transcendental* development and adaptation, following the triadic structure: thesis-antithesis→synthesis (Figure 1). The coupling: ***B not A***, is qualitatively different from ***A*** and ***B*** as constituent components of that coupling. *B* and *A* are integrated (synthesized), forming an *emergent* quality of existence because “the given makes *itself* evident as a *lack*” (Kosok, 1976, p. 328). Furthermore, each negation is the source of a new complex and higher level of existence, which had not existed previously (a meta-level).

Vygotsky himself was “engaged by the Hegelian formula ‘thesis, antithesis, synthesis…” (Vygotsky, 1986, p. xii), and the dialectical method that he incorporates “owes much to Hegel’s dialectic concept, which was later used by Marx and Engels” (Gajdamaschko, 2011, p. e97). In some studies, the dialectic has been mistreated by omitting the contradiction (e.g., Ziemke and Sharkey’s, 2001; Zlatev, 2001; Crowder and Friess, 2010; Tang et al., 2020). However, even when contradiction was noted, it was still mistreated. Costa and Martins (2016) disintegrated the contradiction by choosing only one of its contradictories, unlike the dialectical unification of contradictories. Additionally, those authors considered a contradiction to be an undesired moment. He et al. (2017) considered the *antithesis* to being a real absence of the *thesis*, in contrast to the dialectical *positive absence* (Kosok, 1966). This view is an idealist position in that it adopts one side and excludes the other (Dafermos, 2014). In Hegel’s words regarding zero, “the non-existence of something is a *specific* non-existence, i.e., in the end, it is a real non-existence” (Vygotsky, 1997, p. 249). On the other hand, some researchers have stressed the role of contradiction in productivity. However, they have adopted the concept from “TRIZ” (the Russian abbreviation of inventive problem-solving theory) and not from the system of dialectic logic (see e.g., Mizuyama and Ishida, 2007; Lim et al., 2018). Therefore, such studies omitted the epistemological and methodological role of contradiction. In turn, Crowder and Friess (2013) referred to the role of paradox and conflict in the system from a dialectical position only in words. A primary implementation of contradiction-based meaning can be found in El Maouch et al. (2019a,b, c).

### More on the Formalization of the Emergence of Contradiction-Based Meanings

This section discusses the ways in which contradiction-based meaning involves numerous mental processes, including element fusion, concept formation and language acquisition, abstraction and generalization, attention and active perception, and even including higher needs and subjective skills (such as curiosity and active learning). We borrow Ziemke and Sharkey’s (2001, p. 721) example, which was in turn adopted from the discussion of Zlatev (2001) regarding the meaning of an obstacle, wherein an agent is trying to move forward, but an object is blocking the way. A contradiction is between desired state *A* (*to move forward* or *not to be blocked*) and current state *B* (an object is blocking the movement). A component of *A* is the desired *D. B’s* components are as follows: moving forward action *F*, sensory inputs *S*: the touch sensors, and the image of the blocking object. In addition, a negative emotion *E*^*i*(–)^ emerges because *D* is not satisfied. Despite the skeptical view of artificial emotions (e.g., Searle’s, 1980), emergent emotions are meaning-based. This situation differs from mainstream models, according to which emotions lose their functional/intellectual content. Losing the content is because these models are purely neurobiological and sensual, following the connectionist framework and reinforcement learning (simple positive and negative pulses), or formal, by focusing mainly on facial and bodily appearances (see e.g., Crowder and Friess, 2010; Sequeira et al., 2014; Zhong et al., 2016; Savery and Weinberg, 2020). Emotions are crucial for subjective AI, especially for affective human-robot interaction (HRI) (see e.g., Carter, 2007; Ekbia, 2008; Ziemke and Lowe, 2009; Ziemke, 2016; Dennett, 2017). Emotions represent “a different style of thinking” (Abraham, 2021, p. 3520).

By coupling the contraries in contradiction *Ct*, the meaning *M* of the agent’s experience at this moment *i* becomes *Ct*^*i*^ = *B*(*F* + *S* + *E*^*i*(–)^) *not*(¬) *A*(*D*). The agent grasps the abstract functional relationships among the elements of *A* and *B*: *I am blocked*. In addition, *M*^*i*^ is embodied in the initial components of the contradiction: *D*, *F*, *S*, and *E*^*i*(–)^. Furthermore, since *M*^*i*^ and *E*^*i*(–)^ mediate the agent-environment interaction, the agent perceives the content of *F* and *S* as a precondition (anticipation) of facing an obstacle.

**FIGURE 2:**
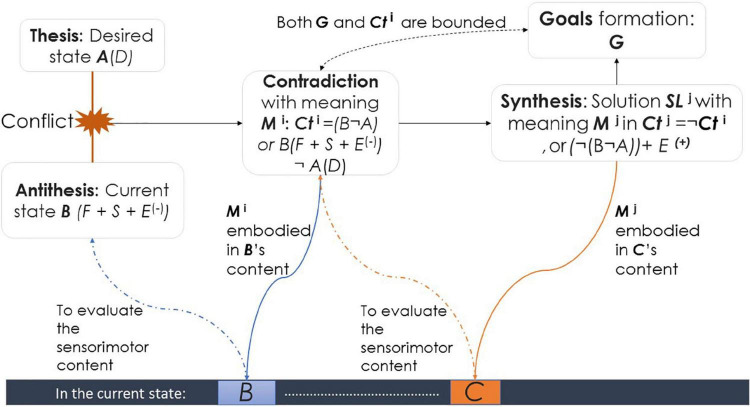
The basic structure of contradiction-based meanings emergence and goals formation.

Later, if in current state *C* and moment *j*, the agent manages to solve *Ct*: *B* not *A*, a solution (a synthesis) *SL* **^*j*^** emerges alongside a positive emotion *E*^*j*(+)^. Thus, *M*^*j*^ becomes ***Ct***^*j*^ = *not Ct* **^*i*^**, or *(¬ (B ¬ A)* + *E*^*j*(+)^. Following the triadic structure: thesis-antithesis synthesis, *Ct*^*i*^ represents the antithesis and *Ct^j^* represents the synthesis (Figure 2). Now, the agent can acquire the concept *free* (or *unblocked)*. Again, *M* ***^j^*** and *E*^*j*(+)^ mediate the agent-environment interaction in perceiving and anticipating a potential solution to the obstacle. By grasping the contradiction (and its solution), the agent represents *interpretive* ability. Moreover, because the content of the solution forms the content of the goal (Leontiev, 1978), the result of *SL^j^* becomes the content of the agent’s goal *G*, which the agent seeks when the next obstacle is faced, hence representing *goal ownership* as an *internal functional switch* related to failure, success, and learning (Bickhard and Terveen, 1996; Haselager, 2005).

In contrast to Gibsonian affordance, as in the mainstream direction, dynamic perception is not only a direct inference concerning what actions can be performed with an object (Albrechtsen et al., 2001). Perception requires attention (Vernon et al., 2016), which is imposed by the selectivity of current meanings (and the problem that is to be solved), because “an eye that would see everything, would for this very reason see nothing” (Vygotsky, 1997, p. 274). In this case, even the absence of the obstacle has meaning (a positive absence). For instance, when the desire is to move forward, the agent needs to avoid facing an obstacle (*not Ct* ***^i^***) following its goal *G*. Therefore, in conditions in which the agent does not face the obstacle, the agent perceives the environment as having *M*^*j*^.

Furthermore, when the agent can reflect several objects that have the same function (here, it is ***not A***), the agent generalizes the acquired knowledge by classifying all objects sharing this common functionality in one category: *obstacles*.

**FIGURE 3:**
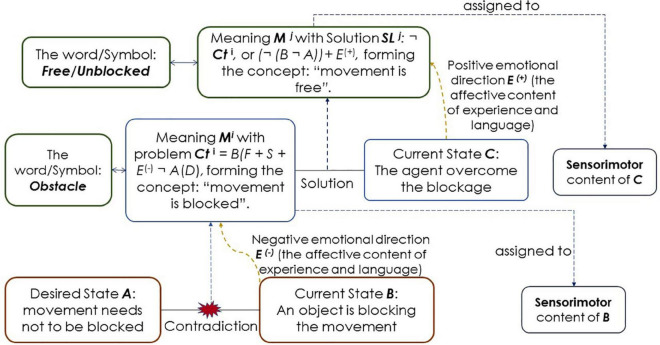
Example of meanings-based concepts and language acquisition.

The above example shows that AI models may solve numerous challenges simultaneously by adopting contradiction-based meaning, such as multisource data fusion, abstraction, generalization, the unification of affect and intellect, concept formation, language acquisition, interpretability, and goal ownership from the context of the self-perspective error, among others (also see Section “The Current Debate: The Central *Problems*”). Regarding concept formation, the content of *M^*i*^* and *M^j^* become the content of the concepts *obstacle* and *free/unblocked*, respectively, with the functional (semantic) content: *movement forward is blocked* and *movement is unblocked* (Figure 3). Now, the agent can acquire the words/symbols *obstacle* and *free* not as dead and meaningless symbols, unlike the direct mainstream association among symbols, actions, and the external features of phenomena (Taniguchi et al., 2018). The word is “an act of thought” and a generalization through the unification of meanings and symbols. “Memorizing words and connecting them with objects does not in itself lead to concept formation; for the process to begin, a problem must arise” (Vygotsky, 1986, p. 100). Furthermore, language/speech, as a tool of social communication, *entails sharing abstract content in the context of solving practical and mental contradictions/problems* (Leontiev, 1978). Here, in addition to *grounding*, the word would refer to numerous concepts and gain new content with each experience, hence answering the *mapping problem* (see Ekbia, 2008).

Moreover, *contradiction-based meanings allow for the formalization of complex and abstract higher mental and psychological processes*, such as curiosity, active learning, and complex social functions.

For instance, let us investigate the internal structure of active learning ability or active engagement. This structure must consist of a coupling of two contradictions; what are they?

Let us suppose that the agent has a concrete desire *N*^1^ (e.g., to reach a source of light) and another abstract desire *N*^2^, such that for each emerged perturbation *Ct^x^*, a solution *SL^x^* (not *Ct*^*x*^) is needed: *N*^2^ = **if** *Ct*^*x*^, **obtain** *SL*^*x*^. Therefore, if in a specific current state *CS*^1^, *N*^1^ is not satisfied (*Ct*^1^ = C*S*^1^ not *N*^1^), *N*^2^ is disturbed because a contradiction (*Ct*^1^) does not yet have a solution. Therefore, *Ct*^2^ = not *N*^2^ emerges. Later, let us suppose that in a state *CS*^2^, the agent reaction (movement) *R* facilitates the satisfaction of *N*^1^, hence solving *Ct*^1^, and a solution *SL*^1^ (*CS*^2^ not *Ct*^1^) emerges. As a result, *Ct*^2^ is solved as well, and the solution *SL^2^ (CS*^2^ not *Ct*^2^) emerges. Thus, the robot gains two subjective contents through *SL*^1^ and *SL*^2^. For one thing, *R* can help satisfy the concrete need *N*^1^. In addition, secondly, it can help satisfy the abstract need *N*^2^. Therefore, when any contradiction appears (not only regarding the need to reach the source of light), the agent uses the content of *SL*^2:^ “I have to act through my body to solve the problem that appeared.” *SL*^2^ is the satisfaction of needs of any type. Therefore, *active engagement* occurs when the agent’s action (R) is embodied by the meaning to satisfy one’s own needs: ***if*** *Ct*^2^ (not *N*^2^)→R.

**FIGURE 4:**
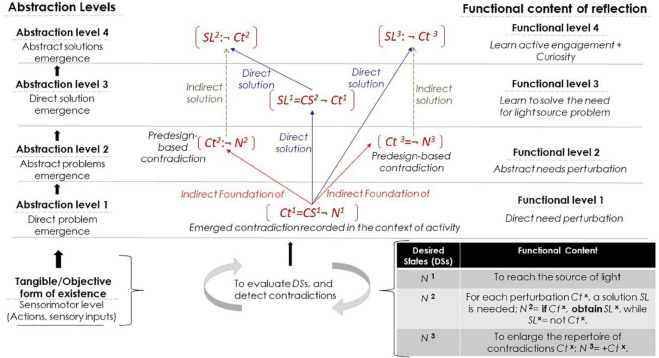
Example of the hierarchy and dependency of developmental levels and how the abstract emerges from the tangible.

Due to space limitations, consider one last example: *curiosity*. Regarding *curiosity*, we introduce an abstract need *N*^3^: the need to enlarge the repertoire of contradictions *Ct^x^*; *N*^3^ = + *Ct^x^*. Let us suppose that the direct need *N*^1^ (to reach the source of light) enters an unsatisfied state after some time. Therefore, *Ct*^1^ = not *N*^1^ emerges; hence, the condition of *N*^3^ is fulfilled because we have a new contradiction. Therefore, the solution *SL*^3^ of *Ct*^3^ (not *N*^3^) also emerges. *SL*^3^ is embodied in action *A*, which is part of forming *Ct*^1^. *A* gains the functional meaning: *my action leads me to gain new problems*. This meaning is the content of *curiosity*. Curiosity leads to an exploration of (moves in) the environment in search of new contradictions to satisfy the need *N*^3^. Furthermore, the word *curious* can be acquired (Figure 4).

By providing the *hierarchy of ever more abstract concepts* (see the *flexibility* problem, the *stability-plasticity* dilemma, and the *binding* problem*)* (see Ekbia, 2008), multiple negations overcome the connectionist’s restraints. Furthermore, grasping one’s own experience (the flow of the perturbations/solution-based activity) provides the agent with a crucial narrative tool that enhances and functionally situates the personalization and interaction (see e.g., El Maouch et al., 2019b,c) because “a robot’s narrative allows humans to get an insight into long term human-robot interaction from the robot’s perspective” (Moulin-Frier et al., 2017, p. 4).

## Conclusion

Artificial intelligence (AI) inherited the crisis in psychology, leading to the domination of mind-body duality reflected in empiricist epistemology and resulting in methodological and technical challenges. Following the epistemology and methodology of dialectics and CHAT, this paper introduces the emergence of qualitative contradiction-based meaning. This viewpoint differs from those of formal associationism and mechanistic-quantitative connectionist methodologies. Meaning emergence entails the simultaneous solution of numerous problems. From these problems, we mention the functional reflection of reality, abstraction, and the question of grasping the substance/content of phenomena, generalization, synthesis (fusion) of elements, and the emergence of higher psychosocial abilities such as concept formation, and language acquisition. Additionally, by explaining the functional internal dependency of mental aspects, the unified dynamic of the mind is affirmed in practice.

Furthermore, we do not need a *genuine* biophysiological organism to whiteness the emergence of meanings. Such emergence means to grasp contradictions, transforming the tangible into the abstract through newly emergent qualities in existence. Ultimately, the aim is not to repeat the path of nature and history. Artificial systems are not required to (and cannot) be a copy of animal- or human-like intelligence due to onto/phylogenetic conditions, since, as Vygotsky (1978) noted, to understand an ongoing process is to study the history of that process in action (see Section “More on the Formalization of the Emergence of Contradiction-Based Meanings”).

An AI system may overcome the present challenges by being able to abstract and qualitatively reflect reality (governed by bodily and environmental constraints). Therefore, the crucial aspect is to grasp the contradiction. Furthermore, by introducing predesigned repertoires of contradiction-based meanings the agent may avoid long learning times. Such a strategy differs from current knowledge-based models because we do not provide formal static knowledge; instead, the agent is equipped with abstract processes (and methodologies). Such repertoire is the outcome of analyzing the contradictions forming various mental abilities, as we have done in the context of *active engagement* and *curiosity* (see Figure 4).

The full potential of the proposed approach requires a great deal of dedicated work. However, due to space limitations, we have focused on the ways in which meaning bridges the abstract-tangible gap, in contrast to the empiricist, sensualist, quantitative, and connectionist frameworks dominating the mainstream AI research. The above discussion demonstrates how the applied level in the context of AI may provide a potential answer to the historical debates in psychology (and philosophy). Thus, despite the *destructive* aspects of the crisis, it “reveals the growth of the science, its enrichment, its force, not its impotence or bankruptcy” (Vygotsky, 1997, p. 295). Incidentally, the term *crisis* in Chinese is written by combining two characters: danger (危) and opportunity (機).

## Data Availability Statement

The original contributions presented in the study are included in the article/supplementary material, further inquiries can be directed to the corresponding author/s.

## Author Contributions

ME was principally responsible for the text in terms of epistemological, theoretical, and methodological proposals as well as developing the alternative guidelines. ZJ contributed to the review and the historical investigation and provided methodological and literature suggestions. Both authors contributed to the manuscript’s final form.

## Conflict of Interest

The authors declare that the research was conducted in the absence of any commercial or financial relationships that could be construed as a potential conflict of interest.

## Publisher’s Note

All claims expressed in this article are solely those of the authors and do not necessarily represent those of their affiliated organizations, or those of the publisher, the editors and the reviewers. Any product that may be evaluated in this article, or claim that may be made by its manufacturer, is not guaranteed or endorsed by the publisher.
